# Motor Skill Training Effect on Real-Time Prefrontal Cortex Activation During Walking

**DOI:** 10.1093/geroni/igab046.616

**Published:** 2021-12-17

**Authors:** Andrea Rosso, Nemin Chen, Subashan Perera, Theodore Huppert, Jessie VanSwearingen, Jennifer Brach, Caterina Rosano

**Affiliations:** 1 University of Pittsburgh, Pittsburgh, Pennsylvania, United States; 2 School of Health and Rehabilitation Sciences, Pittsburgh, Pennsylvania, United States; 4 Graduate School of Public Health, University of Pittsburgh, Pittsburgh, Pennsylvania, United States

## Abstract

We aimed to test the effects of motor skill training (MST) on gait automaticity measured by changes in prefrontal cortex (PFC) activation during actual walking. We used data from a 12-week trial of older adults (mean age=75.5, 60.5% women) randomized to standard physical therapy and standard+MST in a 1:1 ratio. Functional near infrared spectroscopy (fNIRS) measured PFC activation during simple and dual task walking. We will apply linear mixed models to assess effects of task, time, and MST on PFC activation. We will compare the PFC activation 1) during dual task walking compared to simple walking; 2) across visits after intervention; and 3) between participants receiving MST compared to standard physical therapy. These results will demonstrate whether gait automaticity, as evidenced by PFC activation during walking, is affected by MST.

